# Associations between social determinants of health and comorbidity and multimorbidity in people of black ethnicities with HIV

**DOI:** 10.1097/QAD.0000000000003848

**Published:** 2024-02-01

**Authors:** Lourdes Dominguez-Dominguez, Lucy Campbell, Birgit Barbini, Julie Fox, Elena Nikiphorou, Louise Goff, Heidi Lempp, Shema Tariq, Lisa Hamzah, Frank A. Post

**Affiliations:** a Department of Sexual Health and HIV, Kings College Hospital NHS Foundation Trust; b HIV Research Group; c Department of Infectious Diseases, King's College London; d Guy's and St Thomas’ Hospital NHS Foundation Trust; e Department of Rheumatology, Kings College Hospital NHS Foundation Trust; f Centre for Rheumatic Diseases; g Department of Nutritional Sciences, King's College London, London; h Leicester Diabetes Research Centre, Leicester; i University College London; j St George's Healthcare NHS Foundation Trust, London, UK.

**Keywords:** co-morbidity, deprivation, economic, HIV, multimorbidity, social

## Abstract

**Objective:**

Social determinants of health (SDH) are important determinants of long-term conditions and multimorbidity in the general population. The intersecting relationship between SDH and multimorbidity in people with HIV remains poorly studied.

**Design:**

A cross-sectional study investigating the relationships between eight socio-economic parameters and prevalent comorbidities of clinical significance and multimorbidity in adults of African ancestry with HIV aged 18–65 years in South London, UK.

**Methods:**

Multivariable logistic regression analysis was used to evaluate associations between SDH and comorbidities and multimorbidity.

**Results:**

Between September 2020 and January 2022, 398 participants (median age 52 years, 55% women) were enrolled; 85% reported at least one SDH and 72% had at least one comorbidity. There were no associations between SDH and diabetes mellitus or kidney disease, few associations between SDH (job and food insecurity) and cardiovascular or lung disease, and multiple associations between SDH (financial, food, housing and job insecurity, low educational level, social isolation, and discrimination) and poor mental health or chronic pain. Associations between SDH and multimorbidity mirrored those for constituent comorbidities.

**Conclusion:**

We demonstrate strong associations between SDH and poor mental health, chronic pain and multimorbidity in people of black ethnicities living with HIV in the UK. These findings highlight the likely impact of enduring socioeconomic hardship in these communities and underlines the importance of holistic health and social care for people with HIV to address these adverse psychosocial conditions.

## Introduction

Social determinants of health (SDH) (comprising a range of social, economic, and environmental factors) are “the conditions in which people are born, grow, live, work and age” which influence health outcomes [1]. They play a central role in driving health inequalities; in the UK, despite free-at-the-point-of-contact healthcare, health outcomes demonstrate a marked social gradient, with the most socio-economically disadvantaged groups having the lowest life expectancy and disability-free life expectancy [2]. Low income and employment, low levels of education, poor quality housing and overcrowding, loneliness and social isolation, ethnicity, and sex have all been demonstrated to contribute to the development of long-term conditions and poor health outcomes [3]. SDH are also associated with multimorbidity, where an individual lives with more than one long-term condition [4]. Multimorbidity [5,6] affects almost one-third of adults in the UK, with a significant impact on healthcare resources, accounting for more than half of primary care appointments and admissions to hospital [7].

People with HIV experience a disproportionate level of intersecting social disadvantages including lower socioeconomic status [8], poor mental health [9], substance misuse [10], structural racism [11], sex-based violence [12], and in particular, HIV-related stigma including self-stigma [13]. The impact of SDH on engagement with HIV care and virological suppression is well recognized; nonwhite ethnicity, low education level, low health literacy, poverty, food insecurity, homelessness, criminal justice involvement, and intimate partner violence have all been associated with suboptimal engagement with care, poor adherence to antiretroviral therapy (ART) and/or viral rebound [14–18].

People with HIV already live with one chronic health condition, and compared with age-matched adults without HIV, are more likely to accumulate additional comorbidities and live fewer disability-free years, despite effective treatment with ART [19]. Age-related comorbidities such as atherosclerotic cardiovascular disease, diabetes, and chronic kidney disease are more prevalent in this population [20,21], may manifest at a younger age and/or follow a more accelerated course than in the general population [22,23], and contribute to an estimated three-fold increase in mortality per additional comorbidity acquired [24]. Poor mental health is also commonly reported by people with HIV [8,25–29].

The drivers of multimorbidity in people with HIV are many and complex. Apart from traditional risk factors (such as smoking, dyslipidemia, obesity, and hypertension), immune dysregulation, chronic inflammation, HIV viraemia, and exposure to (especially older) antiretroviral agents play an important role in pathogenesis of comorbidities in HIV [22,30–33].

Despite the well evidenced links between SDH and multimorbidity, and HIV and multimorbidity, there are limited data on the intersecting relationship between SDH and multimorbidity in people with HIV. Studies often focus on single exposures, such as the association between homelessness or housing insecurity and poor physical or mental health, or the association between low socioeconomic status and frailty [34]. Such approaches fail to attend to the complexity and intersectionality of people's lives and may limit our ability to provide optimal care and support for people experiencing social marginalization.

Drawing upon quantitative cross-sectional data from the Cardiovascular disease, Kidney disease, and Diabetes in people of African ancestry with HIV (CKD-AFRICA) study, we investigated associations between SDH and comorbidity and multimorbidity in people of black ethnicities living with HIV in South London, UK, an area with high socioeconomic deprivation, multimorbidity and HIV prevalence [35,36].

## Materials and methods

This is an analysis of data from the CKD-AFRICA study, conducted to identify clinical and sociodemographic risk factors for cardiovascular disease, diabetes, and kidney disease. People were eligible to participate in the study if they were living with HIV, aged between 30 and 65 years, of self-reported black African, black Caribbean and/or other black ethnicities, and had previously participated in the Genetic Determinants of Kidney Disease in People of African Ancestry with HIV (GEN-AFRICA) study (NCT05685810) at one of three participating study sites across South London. Participation in GEN-AFRICA was open to all adults of black ethnicities with HIV, who attended for HIV follow-up care at one of nine recruiting sites across the UK, and who were able and willing to provide informed consent, demographic and clinical data, and a blood and urine sample for research [37]. All consecutive clinic attendees meeting these criteria were approached at routine clinic visits over a 21-month period and invited to participate.

Between September 2020 and January 2022, attendees were approached at routine clinic visits and invited and consented to participate in a single study visit. Participants were reimbursed £25 for time and travel after all study procedures had been completed. The study was approved by a National Health Service Research Ethics Committee (20/LO/0946) and the Health Research Authority (IRAS 278244) and all participants provided written informed consent.

### Exposure variables

The following parameters were obtained at the study visit; sex at birth, age, anthropometric data (height, weight, and waist circumference) and three standardized blood pressure measurements. HIV parameters (time since HIV diagnosis, being on and time since starting ART, nadir and recent CD4^+^ cell count, HIV RNA, and hepatitis B surface antigen and antihepatitis C status) and other clinical parameters (use of antihypertensive medication, alcohol consumption, smoking status, CRP, HbA1c, and *APOL1* status [0/1/2 variant alleles) were obtained from clinical notes, self-reported medical history, and fasted urine and blood samples.

Data on socioeconomic status and quality of life were measured by self-completion of paper questionnaires (with support when required), using validated tools; Questionnaire for Verifying Stroke-Free Status [38], St George's Respiratory Questionnaire [39], Hospital Anxiety and Depression Scale [40], the Short Discrimination and Stigma Scale [41], Social Functioning Questionnaire [42] and EQ-5D-5L [43]. Data on employment status, highest completed education level, housing status, income, food security, immigration status, caring responsibilities, and social support were obtained [44]. SDH were defined using composite or simple parameters and included financial insecurity, food insecurity, housing insecurity, migration status insecurity, job insecurity, low education status, social isolation, and discrimination (Table 1). Heath care access was not considered as a variable of interest as all UK-based clinics provide high-quality, free at the point of access healthcare and medication for HIV, regardless of migration status.

**Table 1:** Definitions of the social determinants of health.

Variable	Type	Definition
Financial insecurity	Composite	Being behind with some or most bills, or Not having enough money to meet basic needs or having enough money to meet basic needs only some of the time, or Having needed financial support in the last year
Food insecurity	Simple	Having needed food support in the last year
Housing insecurity	Simple	Living in temporary accommodation, living with friends or family (lacking own accommodation), or being homeless
Migration status insecurity	Composite	Having temporary or limited leave to remain, or having no legal status in the UK, or Having needed immigration support in the last year
Job insecurity	Composite	Being unemployed, sick, or disabled, or Having needed employment support in the last year
Low educational status	Simple	The equivalent of O levels (age 16) or less
Social isolation	Composite	Being alone or isolated, defined as feeling often: - a lack of companionship, or - left out or isolated from others, or - lonely, or Having no one to turn to for emotional support, or Having needed support with loneliness/isolation in the last year
Discrimination	Composite	Feeling little or not at all fairly treated - by friends, in dating situations, in starting a family or in their social life in general, or - with regards to housing, education or job opportunities, or - with regards to respect to their privacy or personal security and safety, or Feeling avoided because of the HIV status

### Outcome variables

A full self-reported medical history including previously diagnosed cardiovascular disease, diabetes, kidney, lung, liver disease and cancer as well as any other long-term conditions was obtained. Definitions of individual comorbidities of interest (diabetes, kidney disease, cardiovascular disease, lung disease, poor mental health, and chronic pain) are described in Table 2. These were chosen as prevalent conditions of clinical significance among people of black ethnicities and those living with HIV [45,46].

**Table 2:** Definitions of risk factors, comorbidities, and multimorbidity.

Variable	Type	Definition
Risk factors		
Obesity	Simple	BMI ≥30 kg/m^2^
Systemic hypertension	Composite	Mean of the second and third readings of SBP ≥140 mmHg and/or Mean of the second and third readings of DBP ≥90 mmHg and/or Being on antihypertensive medication
Comorbidities		
Diabetes mellitus	Composite	HbA1c ≥6.5% and/or Being on hypoglycemic medication
Kidney disease	Composite	eGFR <60 ml/min/1.73 m^2^ and/or albumin/creatinine ratio >3 mg/mmol
Cardiovascular disease	Composite	Arterial disease, defined as - Diagnosis of cardiovascular or cerebrovascular accident according to medical history or the stroke questionnaire; or - Diagnosis of congestive cardiac failure, or Venous disease, defined as having been diagnosed with deep venous thrombosis or pulmonary embolism
Lung disease	Composite	Having a diagnosis of asthma, chronic obstructive pulmonary disease, or interstitial lung disease; or Having symptomatic lung disease, i.e.: cough, phlegm, shortness of breath and/or wheezing on several or most days
Chronic pain	Composite	Pain present for >1 year, or Attending pain clinic, or Having moderate, severe, extreme pain resulting in time off work, seeing GP, or requiring analgesia
Poor mental health	Composite	HADS score for anxiety and/or depression >11; or Self-reported moderate, severe, or extreme anxiety and/or depression; or On medication for anxiety, depression, or other psychiatric illnesses
Multimorbidity		
Main definition	Simple	≥2 of diabetes mellitus, kidney disease, cardiovascular disease, lung disease, and a composite of poor mental health and/or chronic pain
Restricted definition	Simple	≥2 of diabetes mellitus, kidney disease, cardiovascular disease, and lung disease
Expanded definition	Simple	≥2 of diabetes mellitus, kidney disease, cardiovascular disease, lung disease, other significant comorbidity (Table S2), excluding poor mental health and chronic pain
Sensitivity definition	Simple	≥2 of diabetes mellitus, kidney disease, cardiovascular disease, lung disease, poor mental health, and chronic pain, each counted as separate comorbidities

BP, blood pressure; eGFR, estimated glomerular filtration rate; HADS, Hospital Anxiety and Depression Score.

Multimorbidity was defined as a simple count of the presence of two or more of the comorbidities of interest (in which poor mental health and chronic pain were a composite comorbidity). An expanded definition of multimorbidity included any other self-reported condition as described in Table 2.

No formal sample size calculation was performed. A pragmatic sample size based on the total number of participants in the GEN-AFRICA study, with the aim of recruiting 75 individuals each with diabetes and kidney disease, and 50 with cardiovascular disease, along with 200 people without any reported or measured comorbidity, was obtained; the total sample size was 400, enriched for some of the outcomes of interest as within the GEN-AFRICA study, the majority of participants did not report multiple comorbidities.

### Statistical analysis

Participant characteristics were described, according to distribution of variables, and multiple correspondence analysis was performed to determine clustering of comorbidities. Logistic regression models were used to describe associations between SDH (financial insecurity, food insecurity, housing insecurity, migration status insecurity, job insecurity, low education status, social isolation, and discrimination) and the individual comorbidities of interest and multimorbidity.

All multivariate models were *a priori* adjusted for age and sex at birth [47]. Additional covariates considered for inclusion included those that were deemed to be clinically relevant, not on the causal pathway and *P* value less than 0.1 in univariable analysis. Where a comorbidity was associated with more than one SDH, separate multivariate models were generated for each SDH to account for collinearity among various SDH.

As poor mental health and chronic pain were noted to be strongly associated with SDH in multiple correspondence analysis (Fig S1), several posthoc sensitivity analyses were performed to examine this relationship. For associations with individual comorbidities, participants with poor mental health and/or chronic pain were excluded. For the multimorbidity analyses, participants with poor mental health and/or chronic pain were excluded and poor mental health and chronic pain were each included as individual comorbidities. Of note, obesity and hypertension were considered risk factors for diabetes and kidney/cardiovascular disease, respectively, and not analyzed as comorbidities or as constituents of multimorbidity.

The approach to the analysis was informed by the syndemics framework, which explicitly recognizes that HIV may cluster with other conditions, interact through social, psychological or biological pathways, and is driven by harmful social conditions [48]. We placed an emphasis on the co-occurrence with various SDH associates with both comorbidity and multimorbidity, rather than looking at only one determinant in isolation. Data were complete for clinical data and near complete for questionnaire data and complete case analysis was applied for missing data. All statistical analyses were performed using R (R Foundation, Vienna, Austria; version 4.2.1).

## Results

A total of 398 participants were enrolled in the study. Of these, 112 (28%) were free of comorbidities; 19% had diabetes, 28% kidney disease, 8% cardiovascular disease, 16% lung disease, 30% poor mental health, 26% chronic pain, and 11% other comorbidities (Table 3, and Table S1); 131 participants (33%) had a single comorbidity and 155 (39%) had two or more comorbidities. Overall, median age was 52 (interquartile range [IQR] 45–57) years, 55% were women and most had longstanding and well controlled HIV on ART (Table 3, and Tables S2–S4). Hypertension and obesity were common (present in 53 and 50%, respectively) and 9·4% were current smokers. Several expected associations between demographic/clinical parameters and comorbidities were noted, including male sex, hepatitis C virus co-infection, BMI, systemic hypertension, and CRP with diabetes; systemic hypertension and diabetes with kidney disease; and smoking status with lung disease (Table S5).

**Table 3:** Demographic, clinical, and social characteristics of the study participants.

	Overall	Female	Male	
*n*	398	218	180	*P* ^a^
Demographics/ HIV parameters				
Age	52 [45,57]	**51 [45, 56]**	**54 [45, 58]**	**0.026**
Region of birth				**<0.001**
Sub-Saharan Africa	288 (72.4)	174 (79.8)	114 (63.3)	
Caribbean	37 (9.3)	10 (4.6)	27 (15.0)	
UK/Other	73 (18.3)	34 (15.6)	39 (21.7)	
HIV acquisition mechanism				0.64
Sex between men and women	336 (87.7)	186 (88.6)	150 (86.7)	
Sex between men	1 (0.3)	0 (0)	1 (0.6)	
Vertical transmission	40 (10.4)	20 (9.5)	20 (1.6)	
Blood products	1 (0.3)	1 (0.5)	0 (0)	
Unknown	5 (1.3)	3 (1.4)	2 (1.2)	
Time since HIV infection diagnosis (years)	14 [10, 18]	14 [10, 18]	14 [9, 18]	0.96
On ART	395 (99.2)	215 (98.6)	180 (100.0)	0.32
Time since starting ART (years)	10 [7, 15]	10 [7, 15]	11 [6, 15]	0.89
HIV RNA ≥200 copies/ml	23 (5.8)	11 (5.0)	12 (6.7)	0.64
Nadir CD4^+^ cell count	161 [70, 276]	138 [52, 266]	180 [81, 283]	0.11
Recent CD4^+^ cell count	548 [372, 749]	544 [362, 750]	562 [384, 746]	0.72
HCV Ab positive	5 (1.3)	3 (1.5)	2 (1.2)	1.00
HBsAg positive	27 (7.2)	17 (8.2)	10 (5.9)	0.50
Risk factors				
Current smoker	36 (9.4)	17 (8.1)	19 (11.0)	0.43
Systemic hypertension	212 (53.3)	111 (50.9)	101 (56.1)	0.35
BMI (kg/m^2^)	30.0 [26.5, 34.2]	**32.2 [27.6, 36.8]**	**28.2 [25.7, 31.3]**	**<0.001**
Waist (cm)	98 [90, 105]	98 [89, 107]	97 [90, 104]	0.29
CRP (mg/l)	2 [1, 5]	**3 [1, 5]**	**2 [1, 3]**	<0.001
*APOL1* gene - number of G1/G2 variant alleles				0.61
0	156 (41.5)	90 (43.7)	66 (38.8)	
1	148 (39.4)	79 (38.3)	69 (40.6)	
2	72 (19.1)	37 (18.0)	35 (20.6)	
Comorbidities				
Diabetes mellitus	74 (18.6)	34 (15.6)	40 (22.2)	0.12
Kidney disease	111 (27.9)	60 (27.5)	51 (28.3)	0.95
Cardiovascular disease	33 (8.3)	18 (8.3)	15 (8.3)	1.00
Lung disease	64 (16.1)	34 (15.6)	30 (16.7)	0.88
Poor mental health	120 (30.2)	70 (32.1)	50 (27.8)	0.41
Chronic pain	105 (26.4)	**74 (33.9)**	**31 (17.2)**	**<0.001**
Other comorbidities	45 (11.3)	27 (12.4)	18 (10.1)	0.56
Number of comorbidities				0.79
None	112 (28.1)	59 (27.1)	53 (29.4)	
Single comorbidity	131 (32.9)	71 (32.6)	60 (33.3)	
Multimorbidity^b^	155 (38.9)	88 (40.4)	67 (37.2)	
Socioeconomic factors				
Financial insecurity	207 (52.3)	118 (54.4)	89 (49.7)	0.41
Food insecurity	83 (22.3)	**54 (26.6)**	**29 (17.2)**	**0.040**
Housing insecurity	44 (11.1)	27 (12.5)	17 (9.5)	0.43
Migration status insecurity	48 (12.3)	27 (12.6)	21 (11.9)	0.96
Job insecurity	113 (28.4)	65 (29.8)	48 (26.7)	0.56
Low educational status	124 (32.0)	74 (35.2)	50 (28.2)	0.17
Loneliness and isolation	129 (32.5)	74 (34.1)	55 (30.6)	0.52
Discrimination and unfair treatment	124 (33.3)	68 (33.7)	56 (32.9)	0.97
Number of SDH reported				0.62
None	68 (14.7)	35 (13.7)	33 (16.0)	
One or more	330 (85.3)	183 (86.3)	147 (84.0)	
HIV status not disclosed to family or friends	88 (22.2)	51 (23.5)	37 (20.6)	0.56

*APOL1*, *Apolipoprotein L1*; ART, antiretroviral treatment; CRP, C-reactive protein; HBsAg, hepatitis B virus surface antigen; HCV Ab, hepatitis C cirus antibody. ^a^Categorical data are described with absolute (n) and relative (%) frequencies and compared with the Chi-squared test (with continuity correction), except HCV Ab frequencies which are compared by Fisher's exact test. Continuous data are described with the median and interquartile range and compared with the Kruskal–Wallis test.

b Defined as two or more comorbidities. Data in bold indicate statistically significant differences between female and male participants.

Social determinants of health were highly prevalent in the study population, with 330 (85%) reporting at least one SDH. Financial insecurity was reported by 52%, and loneliness and social isolation, discrimination, or unfair treatment by one-third; 22% had not disclosed their HIV status to anyone beyond their immediate healthcare providers (Table 3).

The associations between SDH and the comorbidities of interest are presented in Table 4. There were no associations between any SDH, and either diabetes or kidney disease. Job insecurity was associated with cardiovascular disease (adjusted odds ratio [aOR] 3.27, 95% confidence interval [CI] [1.44–7.56]); job insecurity (aOR 2.38 [1.30–4.31]), and food insecurity (aOR 2.38 [1.30–4.31]) were associated with lung disease. In sensitivity analyses restricted to participants without poor mental health or chronic pain, associations remained for both job insecurity and cardiovascular disease (aOR 3.53 [1.00–12.2]) and lung disease (aOR 2.69 [1.02–6.82]) but not for food insecurity and lung disease (aOR 2.07 [0. 67–5.80]).

**Table 4:** Associations between social determinants of health and comorbidities.

	Diabetes mellitus (*N* = 74)						Kidney disease (*N* = 111)			Cardiovascular disease (*N* = 33)					
	Univariate model			Multivariate model^a^			Univariate model			Univariate model			Multivariate model^b^		
Social determinants of health	OR	95%CI	*P*	aOR	95%CI	*P*	OR	95%CI	*P*	OR	95%CI	*P*	aOR	95%CI	*P*
Financial insecurity	1.09	0.66, 1.82	0.73	–	–	–	0.93	0.60, 1.44	0.74	1.37	0.66, 2.91	0.40	–	–	–
Food insecurity	**1.66**	**0.91, 2.94**	**0.09**	1.91	0.96, 3.71	0.06	1.17	0.67, 1.98	0.57	1.48	0.62, 3.25	0.35	–	–	–
Housing insecurity	0.96	0.40, 2.06	0.92	–	–	–	1.54	0.78, 2.94	0.20	0.23	0.01, 1.12	0.16	–	–	–
Migration status insecurity	1.19	0.54, 2.43	0.65	–	–	–	1.10	0.55, 2.11	0.77	1.02	0.29, 2.75	0.97	–	–	–
Job insecurity	1.27	0.73, 2.17	0.39	–	–	–	1.39	0.86, 2.22	0.18	**2.60**	**1.25, 5.37**	**0.009**	**3.27**	**1.44, 7.56**	**0.005**
Low educational level	0.64	0.35, 1.13	0.14	–	–	–	0.89	0.55, 1.43	0.64	1.01	0.44, 2.17	0.98	–	–	–
Social isolation	0.85	0.49, 1.46	0.57	–	–	–	1.00	0.62, 1.58	0.99	0.76	0.33, 1.64	0.51	–	–	–
Discrimination	0.85	0.47, 1.48	0.57	–	–	–	1.18	0.72, 1.90	0.51	1.05	0.47, 2.22	0.90	–	–	–
HIV status not disclosed to family or friends	1.39	0.77, 2.45	0.27	–	–	–	1.46	0.87, 2.41	0.15	0.94	0.37, 2.14	0.89	–	–	–

	Lung disease (*N* = 64)						Poor mental health (*N* = 120)						Chronic pain (*N* = 105)					
	Univariate model			Multivariate model^c^			Univariate model			Multivariate model^d^			Univariate model			Multivariate model^e^		
Social determinants of Health	OR	95%CI	*P*	aOR	95%CI	*P*	OR	95%CI	*P*	aOR	95%CI	*P*	OR	95%CI	*P*	aOR	95%CI	*P*
Financial insecurity	1.21	0.71, 2.09	0.49	–	–	–	**2.90**	**1.85, 4.62**	**<0.001**	**2.86**	**1.82, 4.57**	**<0.001**	**2.01**	**1.27, 3.21**	**0.003**	**1.85**	**1.14, 3.05**	**0.014**
Food insecurity	**1.82**	**0.98, 3.27**	**0.051**	**2.37**	**1.22, 4.53**	**0.009**	**2.07**	**1.25, 3.43**	**0.005**	**2.07**	**1.24, 3.46**	**0.005**	**2.16**	**1.28, 3.63**	**0.004**	**2.29**	**1.29, 4.06**	**0.004**
Housing insecurity	0.49	0.14, 1.26	0.18	–	–	–	**2.36**	**1.24, 4.46**	**0.008**	**2.48**	**1.29, 4.76**	**0.006**	1.50	0.75, 2.89	0.23	–	–	–
Migration status insecurity	0.85	0.34, 1.89	0.72	–	–	–	1.29	0.67, 2.41	0.43	–	–	–	1.17	0.58, 2.24	0.64	–	–	–
Job insecurity	**2.10**	**1.20, 3.65**	**0.008**	**2.38**	**1.30, 4.31**	**0.004**	**3.21**	**2.03, 5.10**	**<0.001**	**3.25**	**2.05, 5.20**	**<0.001**	**2.79**	**1.74, 4.48**	**<0.001**	**2.70**	**1.61, 4.54**	**<0.001**
Low educational level	1.14	0.63, 2.01	0.66	–	–	–	1.28	0.81, 2.02	0.28	–	–	–	**1.66**	**1.04, 2.65**	**0.034**	**1.68**	**1.01, 2.79**	**0.043**
Social isolation	1.30	0.74, 2.26	0.35	–	–	–	**7.75**	**4.83, 12.6**	**<0.001**	**7.88**	**4.89, 12.9**	**<0.001**	**2.45**	**1.54, 3.90**	**<0.001**	**2.75**	**1.66, 4.59**	**<0.001**
Discrimination	1.10	0.60, 1.97	0.76	–	–	–	**2.82**	**1.78, 4.50**	**<0.001**	**2.92**	**1.83, 4.67**	**<0.001**	**2.20**	**1.36, 3.55**	**0.001**	**2.18**	**1.30, 3.67**	**0.003**
HIV status not disclosed to family or friends	0.88	0.44, 1.66	0.70	–	–	–	1.03	0.61, 1.71	0.92	–	–	–	0.98	0.56, 1.66	0.94	1.10	0.50, 2.26	0.80

Data from logistic regression models including all participants.

a Covariates included in the model: sex and age (regardless univariate model results), hepatitis C antibody status, BMI, hypertension, C-reactive protein.

b Covariates included in the model: sex and age (regardless univariate model results), hepatitis B surface antigen status, *Apolipoprotein L1* status, BMI, C-reactive protein.

c Covariates included in the model: sex and age (regardless univariate model results), smoking status, C-reactive protein.

d Covariates included in the multivariate model: Sex and age.

e Covariates included in the multivariate model: Sex, age, hepatitis B surface antigen positive, BMI, and C-reactive protein. Data in bold indicate statistically significant associations.

There were strong associations between SDH and both poor mental health and chronic pain. Social isolation was associated with an almost eight-fold increased odds of poor mental health (aOR 7.88 [4.89–12.9]), and discrimination and job insecurity with three-fold increased odds (aOR 2.92 (4.89–12.9] and 3.23 (2.05–5.20] respectively). Financial insecurity, food insecurity, and housing insecurity also were independently associated with poor mental health. Similar associations were noted with chronic pain, although generally with smaller effect sizes, and an additional association was noted with low education level (Table 4). In sensitivity analyses, while the associations between SDH and poor mental health remained largely unchanged, if analyses were restricted to participants who did not report chronic pain, the associations between SDH and chronic pain were no longer significant when participants with poor mental health were excluded (Table S6).

In the main analysis of multimorbidity (which included diabetes, kidney disease, cardiovascular disease, lung disease, and the composite of poor mental health/chronic pain), significant associations were observed with financial insecurity, food insecurity, job insecurity, social isolation and discrimination, and these persisted after adjustment for demographic, HIV, and other clinical variables (Table 5). In the sensitivity analyses of both the restricted definition of multimorbidity (similar to the main analysis but excluding poor mental health and/or chronic pain) and the expanded definition of multimorbidity (which included diabetes, kidney disease, cardiovascular disease, lung disease, cancer, liver disease and other significant comorbidities but also excluded poor mental health and/or chronic pain), only food insecurity and job insecurity were associated with multimorbidity. Finally, when poor mental health and chronic pain were considered separate comorbidities (and thus a person with both poor mental health and chronic pain was considered to have multimorbidity irrespective of other long-term conditions), significant associations between financial insecurity, food insecurity, job insecurity, social isolation, discrimination, as well as immigration status insecurity, and multimorbidity were observed (Table 5).

**Table 5:** Associations between social determinants of health and multimorbidity.

	Main definition (*N* = 123)^a^						Restricted definition (*N* = 66)^c^					
	Univariate model			Multivariate model^b^			Univariate model			Multivariate model^d^		
Social determinants of health	OR	95% CI	*P*	aOR	95% CI	*P*	OR	95% CI	*P*	aOR	95% CI	*P*
Financial insecurity	**1.80**	**1.17, 2.80**	**0.008**	**1.97**	**1.20, 3.30**	**0.009**	1.08	0.63, 1.85	0.78	–	–	–
Food insecurity	**2.04**	**1.22, 3.37**	**0.006**	**2.32**	**1.27, 4.25**	**0.006**	**1.92**	**1.04, 3.47**	**0.034**	**2.35**	**1.12, 4.86**	**0.022**
Housing insecurity	1.30	0.66, 2.48	0.43	–	–	–	0.61	0.20, 1.48	0.32	–	–	–
Migration status insecurity	1.56	0.83, 2.90	0.16	–	–	–	0.87	0.34, 1.93	0.75	–	–	–
Job insecurity	**2.43**	**1.54, 3.85**	**<0.001**	**3.17**	**1.86, 5.46**	**<0.001**	**2.14**	**1.23, 3.69**	**0.006**	**2.90**	**1.49, 5.69**	**0.002**
Low educational level	1.09	0.68, 1.72	0.72	–	–	–	0.88	0.48, 1.55	0.66	–	–	–
Social isolation	**1.97**	**1.26, 3.08**	**0.003**	**2.94**	**1.74, 5.05**	**<0.001**	0.81	0.45, 1.43	0.48	–	–	–
Discrimination	**1.88**	**1.19, 2.97**	**0.007**	**2.72**	**1.58, 4.75**	**<0.001**	0.76	0.40, 1.37	0.37	–	–	–
HIV status not disclosed to family or friends	1.05	0.63, 1.74	0.85	–	–	–	0.94	0.48, 1.74	0.84	–	–	–

	Expanded definition (*N* = 79)^e^						Sensitivity analysis (*N* = 145)^g^					
	Univariate model			Multivariate model^f^			Univariate model			Multivariate model^h^		
Social determinants of health	OR	95% CI	*P*	aOR	95% CI	*P*	OR	95% CI	*P*	aOR	95% CI	*P*
Financial insecurity	1.31	0.80, 2.18	0.29	–	–	–	**2.11**	**1.39, 3.22**	**<0.001**	**2.35**	**1.46, 3.84**	**<0.001**
Food insecurity	**1.83**	**1.03, 3.21**	**0.037**	**2.34**	**1.21, 4.48**	**0.011**	**2.13**	**1.29, 3.50**	**0.003**	**2.38**	**1.34, 4.25**	**0.003**
Housing insecurity	0.88	0.37, 1.88	0.75	–	–	–	1.36	0.71, 2.55	0.35	–	–	–
Migration status insecurity	0.95	0.41, 1.97	0.89	–	–	–	**1.72**	**0.93, 3.17**	**0.079**	**2.11**	**1.05, 4.27**	**0.036**
Job insecurity	**2.30**	**1.37, 3.83**	**0.001**	**3.11**	**1.72, 5.66**	**<0.001**	**2.81**	**1.80, 4.41**	**<0.001**	**3.74**	**2.23, 6.38**	**<0.001**
Low educational level	0.70	0.39, 1.20	0.20	–	–	–	1.19	0.77, 1.85	0.43	–	–	–
Social isolation	0.95	0.55, 1.60	0.86	–	–	–	**2.91**	**1.88, 4.51**	**<0.001**	**4.64**	**2.77, 7.93**	**<0.001**
Discrimination	1.13	0.66, 1.93	0.65	–	–	–	**2.18**	**1.39, 3.40**	**<0.001**	**2.95**	**1.76, 5.02**	**<0.001**
HIV status not disclosed to family or friends	1.05	0.57, 1.85	0.88	–	–	–	0.99	0.60, 1.61	0.97	–	–	–

Data from logistic regression models. *APOL1*, *Apolipoprotein L1*; ART, antiretroviral treatment; CRP, C-reactive protein; CVD, cardiovascular disease; DM, diabetes mellitus.

a ≥2 of 5 comorbidities [DM, kidney disease, CVD, lung disease, composite of poor mental health and/or chronic pain] (*n* = 123 outcomes).

b Covariates included in the model: sex and age (regardless univariate model results), region of birth, time since HIV diagnosis, time on ART, HIV RNA ≥200 cp/mL, *APOL1* status, smoking status, BMI, systemic hypertension, CRP.

c ≥2 of 4 comorbidities [DM, kidney disease, CVD, lung disease; poor mental health and/or chronic pain not included] (*n* = 66 outcomes).

d Covariates included in the model: sex and age (regardless univariate model results), time since HIV diagnosis, time on ART, *APOL1* number of risk alleles, smoking status, BMI, systemic hypertension, CRP.

e ≥2 of 6 comorbidities [DM, kidney disease, CVD, lung disease, and any other significant comorbidity (as per Table S2); poor mental health and/or chronic pain not included] (*n* = 79 outcomes).

f Covariates included in the model: sex and age (regardless univariate model results), time since HIV diagnosis, smoking status, BMI, systemic hypertension, CRP.

g ≥2 of 6 comorbidities [DM, kidney disease, CVD, lung disease, and poor mental health and chronic pain (considered as separate comorbidities] (*n* = 145 outcomes).

h Covariates included in the model: sex and age (regardless univariate model results), region of birth, time since HIV diagnosis, time on ART, current CD4 cell count, *APOL1* status, smoking status, BMI, systemic hypertension, CRP. Data in bold indicate statistically significant associations.

## Discussion

In this analysis of data from 398 people of black ethnicities with HIV in South London, 72% of whom had at least one comorbidity, we found a high prevalence of socioeconomic deprivation, with 85% reporting at least one adverse SDH. Poor mental health, chronic pain, and multimorbidity were associated with multiple SDH including financial insecurity, food insecurity, job insecurity, social isolation, and discrimination. We found no evidence of an association between SDH and diabetes or kidney disease. These data suggest that comorbidities are variably associated with SDH, or that SDH may variably affect the development of comorbidities and multimorbidity.

We report a strong association between poor mental health in people with HIV and SDH including social isolation, discrimination, and job, financial, food and housing insecurity. Socioeconomic inequalities such as debt [49], lower education level, unemployment [50], and low income [51] are well recognized drivers of poor mental health in the general population. A systematic review of people with HIV in the USA noted multiple social determinants of health to be associated with depression, such as income, housing instability, food insecurity, educational attainment, transactional sex, and low social support [52]; however, individual studies lacked the broad approach used in this study. Syndemic approaches illustrate the complex, mutually reinforcing and converging relationships between SDH and mental health; the Women's Interagency HIV study (WIHS) demonstrated the negative and intersecting influences of depression, food insecurity, violence, and substance misuse on poor HIV outcomes and as drivers for intimate partner violence [53] and highlight the complexity of the interactions between mental health and SDH. Of note, reported substance misuse was extremely low in our cohort; none reported IDU and only 3% alcohol consumption more than 12 units per week, therefore we were unable to explore a possible relationship between substance misuse, mental health, and SDH.

We also report an association between chronic pain and lower education level, social isolation, discrimination, and job, financial, food and housing insecurity. These effect sizes were smaller than those seen for mental health. It is recognized that few studies focus on pain in marginalized populations [54]. In people with HIV, most focus on single SDH with pain noted to be associated with both unemployment [55] and HIV-related stigma [56,57]. Excluding those with poor mental health from our analyses removed the associations with chronic pain and SDH, supporting the notion that these comorbidities are clustered. Studies exploring the link between HIV stigma and pain and depression are conflicted with some reporting a mediating effect of depression [58], while others found no strong association [56].

There are multiple studies linking pain and depression in the general population [59–61]. The link between poor mental health and chronic pain is likely to be complex. For instance, the relationship between depression and chronic pain is bi-directional with possible shared neural mechanisms [62,63]; depression is a predictive factor for chronic pain, and *vice versa*[64]. Prior studies in HIV have noted the overlap of mental and physical health conditions [65], with pain reported in almost a quarter of those with mental health disorders in the Canadian HIV Women's Sexual and Reproductive Health Cohort Study [66]. However, unlike our study, there are few studies that have systematically examined the role of a broad range of discrete SDH on these outcomes in people with HIV [66].

We did not find an association between SDH and either diabetes or kidney disease, in contrast to data in the general population [67,68]. A lack of association between SDH and diabetes and kidney disease could be a result of regular monitoring and preventive healthcare during routine HIV follow-up or could also reflect a bigger role of other risk factors such as ART, which we were unable to investigate in this analysis or may reflect that this study was underpowered to detect a difference [69,70].

In our analysis, social isolation, discrimination and financial, food, and job insecurity, were associated with multimorbidity; however, only job and food insecurity remained significantly associated with multimorbidity when the analysis was restricted to those without poor mental health and/or chronic pain. This suggests that some of the associations between SDH and multimorbidity are partially mediated by poor mental health and pain and highlights the importance of job and food security in relation to health in HIV. Food insecurity has previously been associated with poor health in HIV, with underlying mechanisms including both nutrition and the effects of food scarcity on mental health [71].

The underlying mechanisms driving the relationship between individual SDH and comorbidity or multimorbidity in people with HIV warrants further research to study mutually reinforcing interactions between SDH and biological factors and causal pathways within the syndemics model of health [48]. It is likely that differing and interacting pathways drive adverse physical outcomes. For example, “weathering,” accelerated aging related to structural barriers, material hardship, and systematic racism in marginalized ethnic groups [72] may play an important role. Persistent stressors are hypothesized to drive adverse outcomes through high allostatic loads, inflammation, and/or accelerated epigenetic aging [73–75].

Aside from SDH, it is important to acknowledge the role of biological pathways in the development of comorbidity and multimorbidity. Several studies have shown chronic inflammation and immune activation, which can occur in the setting of controlled viraemia under optimal ART, to be associated with non-AIDS related long-term conditions [30,76,77]. Ashworth *et al.* found obesity, hypertension, and smoking to be stronger determinants of multimorbidity than measures of social deprivation [78], although this should be interpreted cautiously due to the mediating effects of SDH on smoking and BMI [79].

### Strengths and limitations

Strengths of this study include the utilization of a broad spectrum of SDH in a well characterized cohort of people of African ancestry with HIV in South London, one of the most ethnically diverse and socioeconomically deprived areas in the UK. We were able to include mental health and chronic pain in our analyses, which are often excluded as conditions or risk factors [80] and included measures of HIV-related stigma and generalized discrimination as SDH.

However, our study also has several limitations. SDH are complex, intersecting, and synergistic [79], and both comorbidities and SDH occur in clusters [81]. Therefore, associations between individual SDH and individual comorbidities need to be interpreted with caution. The cross-sectional design of our study limits our ability to infer causality, and in this study, we do not focus on the clustering of SDH. Longitudinal studies are required to ascertain the role of SDH in the incidence of co- and multimorbidity in HIV, and studies would benefit from a syndemic approach.

There are no generally agreed or validated definitions for the SDH examined in this study, nor for multimorbidity, which impedes cross-study comparisons. As the study was exclusively performed in people of African ancestry with HIV, our results cannot not be extrapolated to different populations with HIV, or to people of African ancestry without HIV. In addition, despite free at the point of access to healthcare, we were also not able to fully exclude the influence of structural racism and access to healthcare [82] or the influence of ethnicity on SDH. The study was conducted during the COVID-19 pandemic, which may have affected the SDH status of some participants. As the present study was restricted to GEN-AFRICA participants, people with variable or poor engagement in HIV care may be underrepresented in the present analyses.

Finally, data collection relied on self-reported questionnaire data. It is possible that systematic differences in cognitive appraisal exist between individuals with or without comorbidities such as poor mental health or chronic pain, or those with adverse SDH [83]. In the USA, those with a lower educational background were more vulnerable to stress and more likely perceive daily stressors as a greater risk to their financial and self-worth then those with a higher socioeconomic status [84]. We included educational status as a covariate but cannot exclude the influence of other unmeasured confounding or bias.

## Conclusion

We demonstrate a strong association between social determinants of health and poor mental health, chronic pain and multimorbidity in people of black ethnicities living with HIV in south London. The complex relationship between SDH, pain, and mental health warrants further research; our analysis of qualitative data is ongoing, which will allow us to explore the complex processes underlying these relationships, as well as describing lived experiences. However, these current findings highlight the likely impact of enduring socioeconomic hardship in these communities and underlines the importance of holistic health and social care for people with HIV to address these adverse psychosocial conditions.

## Acknowledgements

The authors would like to thank the study participants, members of the CKD-AFRICA study group (Guy's and St Thomas’ Hospital, London: Julie Fox [PI], Anele Waters; King's College Hospital, London: Frank Post [CI], Rachel Hung, Laura Cechin, Vlad Kolodin, Beatriz Santana-Suarez, Leigh McQueen, Lucy Campbell, Bee Barbini, Emily Wandolo, Kate Childs, Sarah Barber); St. Georges University Hospitals, London: Lisa Hamzah [PI], Katie Toler); Africa Advocacy Foundation: Denis Onyango), and to Dr Cheryl Winkler for providing *APOL1* genotype for the study participants and supporting the GEN-AFRICA study.

This study was presented in abstract form at the HIV Drug Therapy conference (HIV Glasgow 2022).

The study was designed by L.C., L.G., H.L., and F.A.P., with input from the community. J.F., L.H., and F.A.P. were site principal investigators and coordinated recruitment and data collection at their sites. L.C. and B.B. assisted with logistic and governance aspects. L.D.-D. performed the analyses with input from L.H. and F.A.P. L.D.-D., E.N., L.G., H.L., S.T., L.H., and F.A.P. interpreted the findings. L.D.D. and L.H. wrote the first draft of the manuscript with input from E.N., L.G., H.L., and S.T. L.D.-D., L.C., B.B., and F.A.P. verified the underlying data. All authors revised and approved the final version of the manuscript.

The database contains personal and sensitive information and is therefore not publicly available. Access to the study data and/or samples is governed by the National Health Service data access policy and those of King's College Hospital NHS Foundation Trust, the study sponsor. The Gen-AFRICA and CKD-AFRICA studies are open to collaborations, and all requests from researchers who meet the criteria for access to fully anonymized patient level data will be considered. Concepts can be submitted for review to the principal investigator (Prof. Frank Post; E-Mail: frank.post@kcl.ac.uk).

The GEN-AFRICA study was supported by the Medical Research Council (UK) Confidence in Concept scheme (MC_PC_17164), and the CKD-AFRICA study by the Guy's and St Thomas’ Charity Multiple Long Term Conditions Challenge Fund (EIC180702). The funders had no input in the design or conduct of the study, and were not involved in writing of the manuscript or the decision to publish our findings.

### Conflicts of interest

Dr Post reports grants and personal fees and nonfinancial support from Gilead, grants and personal fees and nonfinancial support from ViiV, grants and personal fees and nonfinancial support from MSD during the conduct of the study. There are no conflicts of interest for the remaining authors.

## Supplementary Material
