# Supplementary material for: Associations between social determinants of health and comorbidity and multimorbidity in people of black ethnicities with HIV
Source: AIDS. 2024 Feb 1;38(6):835–46. doi: 10.1097/QAD.0000000000003848 (PMC10994070; doi:10.1097/QAD.0000000000003848)
Supplement: Supplementary file 1 [file aids-38-835-s001.docx]

**Supplementary data**

**Table S1: Additional comorbidities analysed in the study population.**

| **Comorbidity** | **Criteria for consideration as active comorbidity** |
| --- | --- |
| **Liver disease** | Cirrhosis or liver transplant |
| **Cancer** | Any diagnosis (not cervical, anal or vulval intra-epithelial neoplasia) |
| **Inflammatory Bowel Disease** | Any diagnosis |
| **Epilepsy** | If poorly controlled with seizures or requiring medication |
| **Endometriosis** | Any diagnosis |
| **Cognitive impairment** | Any diagnosis |
| **Psoriasis** | If requiring treatment |
| **Pituitary adenoma** | If requiring treatment |
| **Peripheral neuropathy** | If requiring treatment |
| **Rheumatological disease** | Any diagnosis (except fibromyalgia) |
| **Sleep apnoea** | Any diagnosis |
| **Spinal disease** | If symptomatic or requiring treatment |
| **Vertigo** | If symptomatic or requiring treatment |

**Table S2: Demographic, clinical, and social characteristics by comorbidity status.**

|  | **Overall** | **Diabetes mellitus** | | **p value^1^** | **Kidney disease** | | **p**  **value^1^** | **Cardiovascular disease** | | **p**  **value^1^** |
| --- | --- | --- | --- | --- | --- | --- | --- | --- | --- | --- |
|  |  | No | Yes |  | No | Yes |  | No | Yes |  |
| n | 398 | 324 | 74 |  | 287 | 111 |  | 365 | 33 |  |
| ***DEMOGRAPHICS / HIV PARAMETERS*** | | | | | | | | | | |
| Sex: Female | 218 (54.8) | 184 (56.8) | 34 (45.9) | 0.12 | 158 (55.1) | 60 (54.1) | 0.95 | 200 (54.8) | 18 (54.5) | 1.00 |
| Age | 52 [45, 57] | **51 [45, 56]** | **55 [50, 59]** | **<0.001** | **51 [45, 57]** | **54 [49, 57]** | **0.006** | 52 [45, 57] | 55 [50, 59] | **0.007** |
| Region of birth |  |  | | 0.09 |  | | 0.91 |  | | 0.79 |
| Sub-Saharan Africa | 288 (72.4) | 234 (72.2) | 54 (73.0) |  | 206 (71.8) | 82 (73.9) |  | 263 (72.1) | 25 (75.8) |  |
| Caribbean | 37 (9.3) | 26 (8.0) | 11 (14.9) |  | 27 (9.4) | 10 (9.0) |  | 35 (9.6) | 2 (6.1) |  |
| UK / Other | 73 (18.3) | 64 (19.8) | 9 (12.2) |  | 54 (18.8) | 19 (17.1) |  | 67 (18.4) | 6 (18.2) |  |
| Time since HIV infection diagnosis (years) | 14 [10, 18] | 14 [10, 18] | 15 [9, 18] | 0.60 | 13 [9, 18] | 15 [11, 18] | 0.09 | 14 [9, 18] | 16 [12, 19] | 0.22 |
| On ART | 395 (99.2) | 321 (99.1) | 74 (100) | 0.93 | 286 (99.7) | 109 (98.2) | 0.39 | 362 (99.2) | 33 (100) | 1.00 |
| Time since starting ART (years) | 10 [7, 15] | 10 [7, 14] | 11 [7, 15] | 0.61 | 10 [6, 14] | 11 [7, 15] | 0.17 | 10 [7, 14] | 11 [8, 15] | 0.51 |
| HIV RNA ≥200 copies/mL | 23 (5.8) | 17 (5.2) | 6 (8.1) | 0.50 | 14 (4.9) | 9 (8.1) | 0.32 | 22 (6.0) | 1 (3.0) | 0.75 |
| Nadir CD4 cell count | 161 [70, 276] | 153 [68, 268] | 174 [72, 303] | 0.30 | 151 [71, 268] | 181 [54, 284] | 0.39 | 162 [66, 275] | 146 [73, 283] | 0.88 |
| Recent CD4 cell count | 548 [372, 749] | 514 [367, 740] | 623 [448, 761] | 0.08 | **500 [346, 736]** | **635 [457, 752]** | **0.003** | 561 [376, 749] | 475 [320, 735] | 0.64 |
| HCV Ab positive | 5 (1.3) | 2 (0.7) | 3 (4.5) | 0.06 | 4 (1.5) | 1 (1.0) | 1.00 | 5 (1.5) | 0 (0.0) | 1.00 |
| HBsAg positive | 27 (7.2) | 22 (7.1) | 5 (7.4) | 1.00 | 19 (7.0) | 8 (7.5) | 1.00 | 22 (6.4) | 5 (15.6) | 0.11 |
| ***RISK FACTORS*** | | | | | | | | | | |
| Current smoker | 36 (9.4) | 29 (9.3) | 7 (9.9) | 1.00 | 23 (8.4) | 13 (12.0) | 0.36 | 32 (9.1) | 4 (12.5) | 0.76 |
| Systemic hypertension | 212 (53.3) | **157 (48.5)** | **55 (74.3)** | **<0.001** | **134 (46.7)** | **78 (70.3)** | **<0.001** | 191 (52.3) | 21 (63.6) | 0.29 |
| BMI (kg/m^2^) | 30.0 [26.5, 34.2] | **29.7 [26.4, 33.9]** | **31.9 [28.2, 35.4]** | **0.008** | 29.7 [26.5, 33.9] | 31.1 [26.9, 35.1] | 0.23 | 29.8 [26.5, 34.1] | 32.1 [27.4, 36.4] | 0.19 |
| Waist (cm) | 98 [90, 105] | **96 [87, 104]** | **103 [97, 111]** | **<0.001** | 97 [90, 105] | 98 [89, 107] | 0.50 | 97 [90, 105] | 98 [88, 115] | 0.34 |
| CRP (mg/L) | 2 [1, 5] | 2 [1, 5] | 3 [1, 6] | 0.07 | **2.00 [1, 5]** | **3 [1, 6]** | **0.038** | 2 [1, 5] | 4 [1, 9] | 0.13 |
| *APOL1* gene - number of G1/G2 variant alleles |  |  | | 0.26 |  | | 0.95 |  | | **0.042** |
| 0 | 156 (41.5) | 129 (42.0) | 27 (39.1) |  | 113 (41.5) | 43 (41.3) |  | **144 (41.7)** | **12 (38.7)** |  |
| 1 | 148 (39.4) | 124 (40.4) | 24 (34.8) |  | 108 (39.7) | 40 (38.5) |  | **140 (40.6)** | **8 (25.8)** |  |
| 2 | 72 (19.1) | 54 (17.6) | 18 (26.1) |  | 51 (18.8) | 21 (20.2) |  | **61 (17.7)** | **11 (35.5)** |  |
| ***SOCIO-ECONOMIC FACTORS*** | | | | | | | | | | |
| Financial insecurity | 207 (52.3) | 167 (51.9) | 40 (54.1) | 0.83 | 151 (52.8) | 56 (50.9) | 0.82 | 188 (51.6) | 19 (59.4) | 0.51 |
| Food insecurity | 83 (22.3) | 62 (20.5) | 21 (30.0) | 0.12 | 58 (21.6) | 25 (24.3) | 0.67 | 74 (21.7) | 9 (29.0) | 0.48 |
| Housing insecurity | 44 (11.1) | 36 (11.2) | 8 (10.8) | 1.00 | 28 (9.9) | 16 (14.4) | 0.27 | 43 (11.9) | 1 (3.0) | 0.21 |
| Migration status insecurity | 48 (12.3) | 38 (11.9) | 10 (13.9) | 0.80 | 34 (12.0) | 14 (13.1) | 0.91 | 44 (12.3) | 4 (12.5) | 1.00 |
| Job insecurity | 113 (28.4) | 89 (27.5) | 24 (32.4) | 0.48 | 76 (26.5) | 37 (33.3) | 0.22 | **97 (26.6)** | **16 (48.5)** | **0.013** |
| Low educational status | 124 (32.0) | 106 (33.8) | 18 (24.7) | 0.17 | 91 (32.7) | 33 (30.3) | 0.73 | 114 (32.0) | 10 (32.3) | 1.00 |
| Loneliness and isolation | 129 (32.5) | 107 (33.1) | 22 (29.7) | 0.67 | 93 (32.5) | 36 (32.4) | 1.00 | 120 (33.0) | 9 (27.3) | 0.64 |
| Discrimination and unfair treatment | 124 (33.3) | 103 (34.0) | 21 (30.4) | 0.67 | 88 (32.4) | 36 (36.0) | 0.59 | 113 (33.2) | 11 (34.4) | 1.00 |
| HIV status not disclosed to family or friends | 88 (22.2) | 68 (21.1) | 20 (27.0) | 0.34 | 58 (20.3) | 30 (27.0) | 0.19 | 81 (22.3) | 7 (21.2) | 1.00 |

**Table S2 (cont.)**

|  | **Overall** | **Lung disease** | | **p value^1^** | **Poor mental health** | | **p**  **value^1^** | **Chronic pain** | | **p**  **value^1^** |
| --- | --- | --- | --- | --- | --- | --- | --- | --- | --- | --- |
|  |  | No | Yes |  | No | Yes |  | No | Yes |  |
| n | 398 | 334 | 64 |  | 278 | 120 |  | 293 | 105 |  |
| ***DEMOGRAPHICS / HIV PARAMETERS*** | | | | | | | | | | |
| Sex: Female | 218 (54.8) | 184 (55.1) | 34 (53.1) | 0.88 | 148 (53.2) | 70 (58.3) | 0.41 | **144 (49.1)** | **74 (70.5)** | **<0.001** |
| Age | 52 [45, 57] | **51 [45, 56]** | **55 [49, 58]** | **0.017** | 52 [45, 57] | 52 [46, 57] | 0.43 | 52 [45, 56] | 53 [46, 59] | 0.12 |
| Region of birth |  |  | | **0.012** |  | | 0.78 |  | | 0.58 |
| Sub-Saharan Africa | 288 (72.4) | **249 (74.6)** | **39 (60.9)** |  | 203 (73.0) | 85 (70.8) |  | 208 (71.0) | 80 (76.2) |  |
| Caribbean | 37 (9.3) | **25 (7.5)** | **12 (18.8)** |  | 24 (8.6) | 13 (10.8) |  | 29 (9.9) | 8 (7.6) |  |
| UK / Other | 73 (18.3) | **60 (18.0)** | **13 (20.3)** |  | 51 (18.3) | 22 (18.3) |  | 56 (19.1) | 17 (16.2) |  |
| Time since HIV infection diagnosis (years) | 14 [10, 18] | 14 [9, 18] | 15 [10, 19] | 0.34 | 14 [10, 18] | 14 [10, 18] | 0.95 | 14 [9, 18] | 15 [10, 19] | 0.31 |
| On ART | 395 (99.2) | 331 (99.1) | 64 (100) | 1.00 | 276 (99.3) | 119 ( 99.2) | 1.00 | 291 (99.3) | 104 (99.0) | 1.00 |
| Time since starting ART (years) | 10 [7, 15] | 10 [7, 14] | 11 [7, 15] | 0.64 | 11 [7, 15] | 10 [7, 15] | 0.51 | 11 [7, 14] | 10 [7, 15] | 0.62 |
| HIV RNA ≥200 copies/mL | 23 (5.8) | 18 (5.4) | 5 (7.8) | 0.64 | 15 (5.4) | 8 (6.7) | 0.79 | 14 (4.8) | 9 (8.6) | 0.24 |
| Nadir CD4 cell count | 161 [70, 276] | 151 [71, 279] | 185 [43, 242] | 0.84 | 151 [71, 273] | 185 [62, 289] | 0.49 | 158 [64, 281] | 169 [86, 260] | 0.94 |
| Recent CD4 cell count | 548 [372, 749] | 548 [370, 751] | 552 [389, 725] | 0.81 | 535 [372, 751] | 559 [372, 740] | 0.92 | 546 [376, 750] | 548 [362, 734] | 0.84 |
| HCV Ab positive | 5 (1.3) | 4 (1.3) | 1 (1.8) | 1.00 | 3 (1.2) | 2 (1.8) | 1.00 | 4 (1.5) | 1 (1.0) | 1.00 |
| HBsAg positive | 27 (7.2) | 24 (7.5) | 3 (5.4) | 0.77 | 22 (8.4) | 5 (4.4) | 0.25 | 24 (8.7) | 3 (2.9) | 0.09 |
| ***RISK FACTORS*** | | | | | | | | | | |
| Current smoker | 36 (9.4) | **25 (7.7)** | **11 (18.3)** | **0.019** | 24 (9.0) | 12 (10.3) | 0.82 | 24 (8.6) | 12 (11.7) | 0.47 |
| Systemic hypertension | 212 (53.3) | 173 (51.8) | 39 (60.9) | 0.23 | 151 (54.3) | 61 (50.8) | 0.60 | 156 (53.2) | 56 (53.3) | 1.00 |
| BMI (kg/m^2^) | 30.0 [26.5, 34.2] | 29.8 [26.6, 34.0] | 31.0 [25.8, 36.7] | 0.37 | 29.8 [26.4, 33.4] | 30.8 [26.8, 35.4] | 0.37 | **29.6 [26.4, 33.2]** | **31.9 [27.3, 35.6]** | **0.024** |
| Waist (cm) | 98 [90, 105] | 97 [90, 105] | 99 [86, 108] | 0.54 | 98 [90, 104] | 98 [90, 110] | 0.39 | **97 [89, 104]** | **100 [90, 111]** | **0.043** |
| CRP (mg/L) | 2 [1, 5] | 2 [1, 5] | 3.00 [1, 7] | 0.09 | 2 [1, 5] | 3 [1, 5] | 0.13 | **2 [1, 5]** | **3 [1, 5]** | **0.029** |
| *APOL1* gene - number of G1/G2 variant alleles |  |  | | 0.71 |  | | **0.04** |  | | 0.48 |
| 0 | 156 (41.5) | 135 (42.3) | 21 (36.8) |  | **99 (37.8)** | **57 (50.0)** |  | 109 (39.6) | 47 (46.5) |  |
| 1 | 148 (39.4) | 123 (38.6) | 25 (43.9) |  | **114 (43.5)** | **34 (29.8)** |  | 112 (40.7) | 36 (35.6) |  |
| 2 | 72 (19.1) | 61 (19.1) | 11 (19.3) |  | **49 (18.7)** | **23 (20.2)** |  | 54 (19.6) | 18 (17.8) |  |
| ***SOCIO-ECONOMIC FACTORS*** | | | | | | | | | | |
| Financial insecurity | 207 (52.3) | 171 (51.5) | 36 (56.2) | 0.58 | **123 (44.6)** | **84 (70.0)** | **<0.001** | **139 (47.8)** | **68 (64.8)** | **0.004** |
| Food insecurity | 83 (22.3) | 63 (20.4) | 20 (31.7) | 0.07 | **47 (18.2)** | **36 (31.6)** | **0.007** | **51 (18.5)** | **32 (33.0)** | **0.005** |
| Housing insecurity | 44 (11.1) | 40 (12.1) | 4 (6.2) | 0.25 | **23 (8.3)** | **21 (17.6)** | **0.012** | 29 (10.0) | 15 (14.3) | 0.31 |
| Migration status insecurity | 48 (12.3) | 41 (12.6) | 7 (10.9) | 0.88 | 31 (11.4) | 17 (14.3) | 0.54 | 34 (11.8) | 14 (13.6) | 0.77 |
| Job insecurity | 113 (28.4) | **86 (25.7)** | **27 (42.2)** | **0.012** | **58 (20.9)** | **55 (45.8)** | **<0.001** | **66 (22.5)** | **47 (44.8)** | **<0.001** |
| Low educational status | 124 (32.0) | 103 (31.6) | 21 (34.4) | 0.78 | 81 (30.3) | 43 (35.8) | 0.34 | **82 (29.0)** | **42 (40.4)** | **0.044** |
| Loneliness and isolation | 129 (32.5) | 105 (31.5) | 24 (37.5) | 0.43 | **52 (18.8)** | **77 (64.2)** | **<0.001** | **79 (27.1)** | **50 (47.6)** | **<0.001** |
| Discrimination and unfair treatment | 124 (33.3) | 104 (33.0) | 20 (35.1) | 0.88 | **68 (26.2)** | **56 (50.0)** | **<0.001** | **79 (28.6)** | **45 (46.9)** | **0.002** |
| HIV status not disclosed to family or friends | 88 (22.2) | 75 (22.5) | 13 (20.3) | 0.82 | 61 (22.0) | 27 (22.5) | 1.00 | 65 (22.3) | 23 (21.9) | 1.00 |

**Table S2 (cont.)**

^1^Categorical data are described with absolute (n) and relative (%) frequencies and compared with the Chi-squared test (with continuity correction), except HCV Ab frequencies which are compared by Fisher’s exact test.

Continuous data are described with the median and interquartile range and compared with the Kruskal–Wallis test.

ART: antiretroviral treatment. HCV Ab: Hepatitis C Virus antibody. HBsAg: Hepatitis B virus surface antigen. BMI: Body Mass Index. CRP: C-reactive protein. *APOL1*: *Apolipoprotein L1.*

**Table S3: Demographic, clinical, and social characteristics by comorbidity/multimorbidity status.**

|  | **Overall** | **No comorbidities** | **Single comorbidity** | **Multimorbidity^1^** | **p value^2^** |
| --- | --- | --- | --- | --- | --- |
| n | 398 | 112 | 131 | 155 |  |
| ***DEMOGRAPHICS / HIV PARAMETERS*** | | | | | |
| Sex: Female | 218 (54.8) | 59 (52.7) | 71 (54.2) | 88 (56.8) | 0.79 |
| Age | 52 [45, 57] | **48 [42, 54]** | **52 [45, 56]** | **54 [49, 59]** | **<0.001** |
| Region of birth |  | | | | 0.07 |
| Sub-Saharan Africa | 288 (72.4) | 86 (76.8) | 84 (64.1) | 118 (76.1) |  |
| Caribbean | 37 (9.3) | 7 (6.2) | 14 (10.7) | 16 (10.3) |  |
| UK / Other | 73 (18.3) | 19 (17.0) | 33 (25.2) | 21 (13.5) |  |
| Time since HIV infection diagnosis (years) | 14 [10, 18] | **14 [9, 19]** | **13 [9, 16]** | **16 [11, 19]** | **0.003** |
| On ART | 395 (99.2) | 112 (100) | 129 (98.5) | 154 (99.4) | 0.38 |
| Time since starting ART (years) | 10 [7, 15] | **11 [7, 15]** | **9 [6, 13]** | **11 [7, 15]** | **0.032** |
| HIV RNA ≥200 copies/mL | 23 (5.8) | 5 (4.5) | 5 (3.8) | 13 (8.4) | 0.20 |
| Nadir CD4 cell count | 161 [70, 276] | 126 [72, 243] | 161 [65, 275] | 183 [68, 287] | 0.37 |
| Recent CD4 cell count | 548 [372, 749] | 503 [370, 747] | 518 [359, 707] | 607 [393, 751] | 0.15 |
| HCV Ab positive | 5 (1.3) | 1 (1.0) | 2 (1.6) | 2 (1.4) | 0.90 |
| HBsAg positive | 27 (7.2) | 9 (8.4) | 10 (8.1) | 8 (5.5) | 0.60 |
| ***RISK FACTORS*** | | | | | |
| Current smoker | 36 (9.4) | 5 (4.6) | 13 (10.3) | 18 (12.1) | 0.12 |
| Systemic hypertension | 212 (53.3) | **50 (44.6)** | **64 (48.9)** | 98 (63.2) | **0.005** |
| BMI (kg/m^2^) | 30.0 [26.5, 34.2] | **28.6 [25.5, 32.4]** | **30.0 [27.3, 33.6]** | **31.6 [26.5, 35.8]** | **0.011** |
| Waist (cm) | 98 [90, 105] | **95 [85, 102]** | **99 [91, 106]** | **99 [90, 110]** | **0.003** |
| CRP (mg/L) | 2 [1, 5] | **2 [1, 4]** | **2 [1, 4]** | **3 [1, 7]** | **0.006** |
| *APOL1* gene - number of G1/G2 variant alleles |  | | | | 0.36 |
| 0 | 156 (41.5) | 42 (39.3) | 47 (38.2) | 67 (45.9) |  |
| 1 | 148 (39.4) | 47 (43.9) | 53 (43.1) | 48 (32.9) |  |
| 2 | 72 (19.1) | 18 (16.8) | 23 (18.7) | 31 (21.2) |  |
| ***SOCIO-ECONOMIC FACTORS*** | | | | | |
| Financial insecurity | 207 (52.3) | **48 (43.2)** | **62 (47.3)** | **97 (63.0)** | **0.002** |
| Food insecurity | 83 (22.3) | **14 (13.7)** | **22 (17.7)** | **47 (32.2)** | **0.001** |
| Housing insecurity | 44 (11.1) | 6 (5.5) | 18 (13.8) | 20 (12.9) | 0.08 |
| Migration status insecurity | 48 (12.3) | 12 (11.0) | 12 (9.3) | 24 (15.8) | 0.23 |
| Job insecurity | 113 (28.4) | **16 (14.3)** | **34 (26.0)** | **63 (40.6)** | **<0.001** |
| Low educational status | 124 (32.0) | 37 (34.3) | 35 (27.6) | 52 (34.2) | 0.42 |
| Loneliness and isolation | 129 (32.5) | **19 (17.1)** | **38 (29.0)** | **72 (46.5)** | **<0.001** |
| Discrimination and unfair treatment | 124 (33.3) | **26 (24.8)** | **35 (28.0)** | **63 (44.4)** | **0.002** |
| HIV status not disclosed to family or friends | 88 (22.2) | 19 (17.1) | 34 (26.0) | 35 (22.6) | 0.25 |

**Table S3 (cont.)**

^1^≥2 of any comorbidities.

^2^Categorical data are described with absolute (n) and relative (%) frequencies and compared with the Chi-squared test (with continuity correction), except HCV Ab frequencies which are compared by Fisher’s exact test.

Continuous data are described with the median and interquartile range and compared with the Kruskal–Wallis test.

ART: antiretroviral treatment. HCV Ab: Hepatitis C Virus antibody. HBsAg: Hepatitis B virus surface antigen. BMI: Body Mass Index. CRP: C-reactive protein. *APOL1*: *Apolipoprotein L1.*

**Table S4: Demographic, clinical, and social characteristics by multimorbidity status.**

|  | **Overall** | **Multimorbidity**  **(main definition)** | | **p value^1^** | **Multimorbidity**  **(restricted definition)** | | **p value^1^** | **Multimorbidity**  **(expanded definition)** | | **p value^1^** | **Multimorbidity**  **(sensitivity definition)** | | **p value^1^** |
| --- | --- | --- | --- | --- | --- | --- | --- | --- | --- | --- | --- | --- | --- |
|  |  | No | Yes |  | No | Yes |  | No | Yes |  | No | Yes |  |
| n | 398 | 275 | 123 |  | 332 | 66 |  | 319 | 79 |  | 253 | 145 |  |
| ***DEMOGRAPHICS / HIV PARAMETERS*** | | | | | | | | | | | | | |
| Sex: Female | 218 (54.8) | 149 (54.2) | 69 (56.1) | 0.81 | 186 (56.0) | 32 (48.5) | 0.32 | 180 (56.4) | 38 (48.1) | 0.23 | 134 (53.0) | 84 (57.9) | 0.39 |
| Age | 52 [45, 57] | **50 [44, 56]** | **55.00 [50, 59]** | **<0.001** | **51 [44, 56]** | **56 [51, 60]** | **<0.001** | **51 [44, 56]** | **55 [51, 59]** | **<0.001** | **50 [44, 56]** | **54 [49, 59]** | **<0.001** |
| Region of birth |  |  | | 0.15 |  | | 0.18 |  | | 0.12 |  |  | 0.10 |
| Sub-Saharan Africa | 288 (72.4) | 195 (70.9) | 93 (75.6) |  | 242 (72.9) | 46 (69.7) |  | 233 (73.0) | 55 (69.6) |  | 178 (70.4) | 110 (75.9) |  |
| Caribbean | 37 (9.3) | 23 (8.4) | 14 (11.4) |  | 27 (8.1) | 10 (15.2) |  | 25 (7.8) | 12 (15.2) |  | 21 (8.3) | 16 (11.0) |  |
| UK / Other | 73 (18.3) | 57 (20.7) | 16 (13.0) |  | 63 (19.0) | 10 (15.2) |  | 61 (19.1) | 12 (15.2) |  | 54 (21.3) | 19 (13.1) |  |
| Time since HIV infection diagnosis (years) | 14 [10, 18] | **13 [9, 17]** | **16 [11, 19]** | **0.005** | **14 [9, 18]** | **16 [12, 19]** | **0.026** | **14 [9, 18]** | **16 [12, 19]** | **0.025** | **13 [9, 17]** | **16 [11, 19]** | **0.003** |
| On ART | 395 (99.2) | 272 (98.9) | 123 (100) | 0.59 | 329 (99.1) | 66 (100) | 1.00 | 316 (99.1) | 79 (100) | 0.89 | 251 (99.2) | 144 (99.3) | 1.00 |
| Time since starting ART (years) | 10 [7, 15] | **10 [6, 14]** | **12 [8, 15]** | **0.027** | 10 [7, 14] | 12 [8, 16] | 0.09 | 10 [7, 14] | 12 [8, 15] | 0.10 | **10 [6, 14]** | **11 [7, 15]** | **0.049** |
| HIV RNA ≥200 copies/mL | 23 (5.8) | 12 (4.4) | 11 (8.9) | 0.12 | 18 (5.4) | 5 (7.6) | 0.69 | 16 (5.0) | 7 (8.9) | 0.30 | 11 (4.3) | 12 (8.3) | 0.16 |
| Nadir CD4 cell count | 161 [70, 276] | 149 [65, 272] | 184 [74, 289] | 0.29 | 160 [68, 276] | 169 [73, 270] | 0.99 | 158 [65, 277] | 174 [73, 259] | 0.79 | 149 [71, 268] | 184 [54, 289] | 0.41 |
| Recent CD4 cell count | 548 [372, 749] | 515 [366, 745] | 610 [397, 752] | 0.05 | 525 [370, 745] | 599 [417, 752] | 0.21 | 530 [369, 742] | 595 [422, 753] | 0.18 | **515 [367, 714]** | **610 [393, 752]** | **0.048** |
| HCV Ab positive | 5 (1.3) | 3 (1.2) | 2 (1.7) | 1.00 | 4 (1.3) | 1 (1.7) | 1.00 | 4 (1.3) | 1 (1.4) | 1.00 | 3 (1.3) | 2 (1.5) | 1.00 |
| HBsAg positive | 27 (7.2) | 19 (7.3) | 8 (7.0) | 1.00 | 24 (7.6) | 3 (5.0) | 0.66 | 24 (7.9) | 3 (4.1) | 0.38 | 19 (7.9) | 8 (5.8) | 0.59 |
| ***RISK FACTORS*** | | | | | | | | | | | | | |
| Current smoker | 36 (9.4) | 20 (7.5) | 16 (13.6) | 0.10 | 26 (8.1) | 10 (15.9) | 0.09 | 24 (7.8) | 12 (15.8) | 0.06 | 18 (7.4) | 18 (12.9) | 0.12 |
| Systemic hypertension | 212 (53.3) | **128 (46.5)** | **84 ( 68.3)** | **<0.001** | **160 (48.2)** | **52 (78.8)** | **<0.001** | **154 (48.3)** | **58 ( 73.4)** | **<0.001** | **119 (47.0)** | **93 (64.1)** | **0.001** |
| BMI (kg/m^2^) | 30.0 [26.5, 34.2] | **29.6 [26.5, 33.3]** | **31.6 [26.7, 36.3]** | **0.027** | **29.7 [26.4, 33.8]** | **32.1 [27.5, 37.1]** | **0.019** | **29.7 [26.4, 33.7]** | **32.0 [27.4, 36.8]** | **0.029** | **29.4 [26.5, 33.0]** | **31.8 [26.9, 36.1]** | **0.013** |
| Waist (cm) | 98 [90, 105] | **97 [90, 104]** | **99 [90, 110]** | **0.045** | **97 [89, 105]** | **101 [92, 114]** | **0.019** | **97 [89, 104]** | **102 [91, 116]** | **0.008** | **97 [89, 104]** | **99 [90, 110]** | **0.021** |
| CRP (mg/L) | 2 [1, 5] | **2 [1, 4]** | **3 [1, 8]** | **0.001** | **2 [1, 5]** | **3 [1, 7]** | **0.018** | **2 [1, 4]** | **4 [1, 8]** | **0.005** | **2 [1, 4]** | **3 [1, 7]** | **0.001** |
| *APOL1* gene - number of G1/G2 variant alleles |  |  | | 0.09 |  | | **0.018** |  | | **0.030** |  |  | 0.15 |
| 0 | 156 (41.5) | 104 (39.8) | 52 (45.2) |  | **132 (41.8)** | **24 (40.0)** |  | 125 (41.3) | 31 (42.5) |  | 93 (38.9) | 63 (46.0) |  |
| 1 | 148 (39.4) | 112 (42.9) | 36 (31.3) |  | **131 (41.5)** | **17 (28.3)** |  | 127 (41.9) | 21 (28.8) |  | 103 (43.1) | 45 (32.8) |  |
| 2 | 72 (19.1) | 45 (17.2) | 27 (23.5) |  | **53 (16.8)** | **19 (31.7)** |  | 51 (16.8) | 21 (28.8) |  | 43 (18.0) | 29 (21.2) |  |
| ***SOCIO-ECONOMIC FACTORS*** | | | | | | | | | | | | | |
| Financial insecurity | 207 (52.3) | **131 (47.8)** | **76 (62.3)** | **0.011** | 172 (52.0) | 35 (53.8) | 0.89 | 162 (50.9) | 45 (57.7) | 0.35 | **115 (45.6)** | **92 (63.9)** | **0.001** |
| Food insecurity | 83 (22.3) | **47 (18.3)** | **36 (31.3)** | **0.008** | **63 (20.3)** | **20 (32.8)** | **0.048** | 60 (20.1) | 23 (31.5) | 0.05 | **41 (17.4)** | **42 (30.9)** | **0.004** |
| Housing insecurity | 44 (11.1) | 28 (10.3) | 16 (13.0) | 0.53 | 39 (11.9) | 5 (7.6) | 0.43 | 36 (11.4) | 8 (10.1) | 0.91 | 25 (10.0) | 19 (13.1) | 0.44 |
| Migration status insecurity | 48 (12.3) | 29 (10.7) | 19 (15.8) | 0.21 | 41 (12.5) | 7 (11.1) | 0.92 | 39 (12.4) | 9 (11.8) | 1.00 | 25 (10.1) | 23 (16.2) | 0.11 |
| Job insecurity | 113 (28.4) | **62 (22.5)** | **51 (41.5)** | **<0.001** | **85 (25.6)** | **28 ( 42.4)** | **0.009** | **79 (24.8)** | **34 (43.0)** | **0.002** | **52 (20.6)** | **61 (42.1)** | **<0.001** |
| Low educational status | 124 (32.0) | 84 (31.5) | 40 (33.3) | 0.81 | 105 (32.5) | 19 (29.7) | 0.77 | 104 (33.5) | 20 (26.0) | 0.26 | 75 (30.6) | 49 (34.5) | 0.50 |
| Loneliness and isolation | 129 (32.5) | **76 (27.7)** | **53 (43.1)** | **0.004** | 110 (33.2) | 19 (28.8) | 0.58 | 104 (32.7) | 25 (31.6) | 0.96 | **60 (23.8)** | **69 (47.6)** | **<0.001** |
| Discrimination and unfair treatment | 124 (33.3) | **75 (29.0)** | **49 (43.4)** | **0.010** | 107 (34.3) | 17 (28.3) | 0.46 | 98 (32.8) | 26 (35.6) | 0.75 | **65 (27.1)** | **59 (44.7)** | **0.001** |
| HIV status not disclosed to family or friends | 88 (22.2) | 60 (21.9) | 28 (22.8) | 0.95 | 74 (22.4) | 14 (21.2) | 0.97 | 70 (22.0) | 18 (22.8) | 1.00 | 56 (22.2) | 32 (22.1) | 1.00 |

**Table S4 (cont.)**

^1^Categorical data are described with absolute (n) and relative (%) frequencies and compared with the Chi-squared test (with continuity correction), except HCV Ab frequencies which are compared by Fisher’s exact test.

Continuous data are described with the median and interquartile range and compared with the Kruskal–Wallis test.

Multimorbidity definitions:

| **Main definition** | Simple | ≥2 of diabetes mellitus, kidney disease, cardiovascular disease, lung disease, and a composite of poor mental health and/or chronic pain |
| --- | --- | --- |
| **Restricted definition** | Simple | >2 of diabetes mellitus, kidney disease, cardiovascular disease, and lung disease |
| **Expanded definition** | Simple | >2 of diabetes mellitus, kidney disease, cardiovascular disease, lung disease, other significant comorbidity (Table S2), excluding poor mental health and chronic pain |
| **Sensitivity definition** | Simple | ≥2 of diabetes mellitus, kidney disease, cardiovascular disease, lung disease, poor mental health and chronic pain, each counted as separate comorbidities |

ART: antiretroviral treatment. HCV Ab: Hepatitis C Virus antibody. HBsAg: Hepatitis B virus surface antigen. BMI: Body Mass Index. CRP: C-reactive protein. *APOL1*: *Apolipoprotein L1.*

**Table S5: Associations between demographic/clinical variables and various comorbidities.**

|  | **Diabetes mellitus** | | | | | |
| --- | --- | --- | --- | --- | --- | --- |
|  | **Univariate model** | | | **Multivariate model^1,2^** | | |
|  | ***OR*** | ***95%CI*** | ***p value*** | ***aOR*** | ***95%CI*** | ***p value*** |
| ***DEMOGRAPHICS/HIV parameters*** | | | | | | |
| **Sex: Female (vs Male)** | 0.65 | 0.39, 1.07 | **0.09** | **0.48** | **0.24, 0.91** | **0.027** |
| **Age (ref: <40)** |  | | |  | | |
| 40-50 years-old | 1.28 | 0.44, 4.70 | 0.68 | 0.89 | 0.26, 3.62 | 0.86 |
| >50 years-old | **3.29** | **1.26, 11.3** | **0.029** | 1.83 | 0.61, 7.13 | 0.32 |
| **Region of birth: Africa/Caribbean (vs Europe)** | 1.78 | 0.88, 4.00 | 0.13 | — | — | — |
| **Time since HIV diagnosis (per year)** | 1.01 | 0.97, 1.05 | 0.73 | — | — | — |
| **On ART** | Excluded | — | — | — | — | — |
| **ART regimen (as per third drug)** |  | | |  | | |
| NNRTI-based | Ref | — | — | — | — | — |
| bPI-based | 1.44 | 0.70, 2.97 | 0.32 | — | — | — |
| INSTI-based | 2.50 | 1.36, 4.77 | **0.004** | — | — | — |
| **ART regimen (as per backbone)** |  | | |  | | |
| TDF | Ref | — | — | — | — | — |
| Nor TDF or TAF | 1.41 | 0.75, 2.74 | 0.29 | — | — | — |
| TAF | 3.69 | 1.88, 7.43 | **<0.001** | — | — | — |
| **Time on ART (per year)** | 1.01 | 0.96, 1.05 | 0.75 | — | — | — |
| **HIV RNA ≥200 copies/mL** | 1.59 | 0.56, 3.99 | 0.35 | — | — | — |
| **Nadir CD4 cell count (per two-fold increase)** | 1.07 | 0.92, 1.26 | 0.37 | — | — | — |
| **Recent CD4 cell count (per two-fold increase)** | 1.06 | 0.83, 1.41 | 0.65 | — | — | — |
| **HCV Ab positive** | **7.10** | **1.15, 54.7** | **0.034** | **9.14** | **1.30, 78.8** | **0.026** |
| **HBsAg positive** | 1.04 | 0.34, 2.64 | 0.95 | — | — | — |
| ***RISK FACTORS*** | | | | | | |
| ***APOL1* genotype (ref: none)** |  | | |  | | |
| One risk allele | 0.92 | 0.50, 1.69 | 0.80 | — | — | — |
| Two risk alleles | 1.59 | 0.80, 3.12 | 0.18 | — | — | — |
| **Smoking status (ref: never)** |  | | |  | | |
| Current | 1.11 | 0.43, 2.55 | 0.81 | — | — | — |
| Ex | 1.43 | 0.61, 3.09 | 0.38 | — | — | — |
| **BMI (per 1 kg/m^2^ increment)** | **1.04** | **1.00, 1.08** | **0.032** | **1.06** | **1.01, 1.11** | **0.029** |
| **Systemic hypertension** | **3.08** | **1.78, 5.54** | **<0.001** | **1.92** | **1.02, 3.75** | **0.048** |
| **CRP (per 1 mg/L increment)** | **1.06** | **1.01, 1.11** | **0.025** | **1.06** | **1.00, 1.12** | **0.044** |

^1^Food Insecurity also included as covariate. Please see Table 3.

^2^ART regimen not included as covariate.

**Table S5 (cont.)**

|  | **Kidney disease** | | | | | |
| --- | --- | --- | --- | --- | --- | --- |
|  | **Univariate model** | | | **Multivariate model^1,2^** | | |
|  | ***OR*** | ***95%CI*** | ***p value*** | ***aOR*** | ***95%CI*** | ***p value*** |
| ***DEMOGRAPHICS/HIV parameters*** | | | | | | |
| **Sex: Female (vs Male)** | 0.96 | 0.62, 1.49 | 0.86 | 1.23 | 0.76, 2.02 | 0.40 |
| **Age (ref: <40)** |  | | |  | | |
| 40-50 years-old | 1.86 | 0.76, 5.27 | 0.20 | 1.48 | 0.57, 4.41 | 0.45 |
| >50 years-old | **3.23** | **1.40, 8.81** | **0.011** | 1.92 | 0.76, 5.57 | 0.19 |
| **Region of birth: Africa/Caribbean (vs Europe)** | 1.12 | 0.64, 2.04 | 0.70 | — | — | — |
| **Time since HIV diagnosis (per year)** | 1.02 | 0.98, 1.06 | 0.27 | — | — | — |
| **On ART** | 0.19 | 0.01, 2.01 | 0.18 | — | — | — |
| **ART regimen (as per third drug)** |  | | |  | | |
| NNRTI-based | Ref | — |  | — | — | — |
| bPI-based | 0.64 | 0.33, 1.18 | 0.16 | — | — | — |
| INSTI-based | 1.70 | 1.03, 2.85 | **0.040** | — | — | — |
| **ART regimen (as per backbone)** |  | | |  | | |
| TDF | Ref | — |  | — | — | — |
| Nor TDF or TAF | 2.29 | 1.33, 4.07 | **0.004** | — | — | — |
| TAF | 3.73 | 2.00, 7.12 | **<0.001** | — | — | — |
| **Time on ART (per year)** | 1.03 | 0.99, 1.07 | 0.19 | — | — | — |
| **HIV RNA ≥200 copies/mL** | 1.72 | 0.70, 4.05 | 0.22 | — | — | — |
| **Nadir CD4 cell count (per two-fold increase)** | 1.01 | 0.89, 1.16 | 0.82 | — | — | — |
| **Recent CD4 cell count (per two-fold increase)** | **1.32** | **1.03, 1.74** | **0.037** | **1.33** | **1.03, 1.75** | **0.038** |
| **HCV Ab positive** | 0.63 | 0.03, 4.34 | 0.68 | — | — | — |
| **HBsAg positive** | 1.08 | 0.43, 2.47 | 0.86 | — | — | — |
| ***RISK FACTORS*** | | | | | | |
| ***APOL1* genotype (ref: none)** |  | | |  | | |
| One risk allele | 0.97 | 0.59, 1.61 | 0.92 | — | — | — |
| Two risk alleles | 1.08 | 0.58, 1.99 | 0.80 | — | — | — |
| **Smoking status (ref: never)** |  | | |  | | |
| Current | 1.42 | 0.67, 2.89 | 0.34 | — | — | — |
| Ex | 0.57 | 0.22, 1.27 | 0.19 | — | — | — |
| **BMI (effect per 1 kg/m^2^ increment)** | 1.02 | 0.98, 1.05 | 0.35 | — | — | — |
| **Systemic hypertension** | **2.70** | **1.70, 4.35** | **<0.001** | **2.01** | **1.20, 3.40** | **0.008** |
| **Glycaemia status (ref: normoglycaemia)** |  | | |  | | |
| Pre-DM | 1.12 | 0.66, 1.90 | 0.67 | 1.02 | 0.58, 1.77 | 0.95 |
| DM | **4.09** | **2.31, 7.33** | **<0.001** | **3.70** | **2.00, 6.91** | **<0.001** |
| **CRP (per 1 mg/L increment)** | 1.03 | 0.98, 1.07 | 0.27 | — | — | — |

^1^No social determinant of health included as covariate as none with positive association in the univariate analyses. Please see Table 3.

^2^ART regimen not included as covariate.

**Table S5 (cont.)**

|  | **Cardiovascular disease** | | | | | | | | |
| --- | --- | --- | --- | --- | --- | --- | --- | --- | --- |
|  | **Univariate model** | | | | | **Multivariate model^1,2^** | | | |
|  | ***OR*** | ***95%CI*** | | ***p value*** | | ***aOR*** | | ***95%CI*** | ***p value*** |
| ***DEMOGRAPHICS/HIV parameters*** | | | | | | | | | |
| **Sex: Female (vs Male)** | 0.99 | 0.48, 2.05 | | 0.98 | | 0.90 | | 0.37, 2.18 | 0.81 |
| **Age (ref: ≤50)** |  | | | | |  | | | |
| >50 years-old | 1.92 | 0.91, 4.33 | | **0.10** | | 1.75 | | 0.76, 4.30 | 0.20 |
| **Region of birth: Africa/Caribbean (vs Europe)** | 1.01 | 0.43, 2.80 | | 0.98 | | — | | — | — |
| **Time since HIV diagnosis (per year)** | 1.03 | 0.98, 1.09 | | 0.26 | | — | | — | — |
| **On ART** | Excluded | — | — | | | | — | — | — |
| **ART regimen (as per third drug)** |  | | | | |  | | | |
| NNRTI-based | Ref | — | |  | | — | | — | — |
| bPI-based | 0.97 | 0.31, 2.86 | | 0.95 | | — | | — | — |
| INSTI-based | 2.36 | 1.03, 5.89 | | **0.05** | | — | | — | — |
| **ART regimen (as per backbone)** |  | | | | |  | | | |
| TDF | Ref | — | |  | | — | | — | — |
| Nor TDF or TAF | 1.06 | 0.43, 2.66 | | 0.91 | | — | | — | — |
| TAF | 2.46 | 0.99, 6.29 | | **0.05** | | — | | — | — |
| **Time on ART (per year)** | 1.03 | 0.97, 1.09 | | 0.37 | | — | | — | — |
| **HIV RNA ≥200 copies/mL** | 0.49 | 0.03, 2.44 | | 0.49 | | — | | — | — |
| **Nadir CD4 cell count (effect per 2-fold increase)** | 0.94 | 0.78, 1.16 | | 0.56 | | — | | — | — |
| **Recent CD4 cell count (effect per 2-fold increase)** | 0.94 | 0.69, 1.36 | | 0.70 | | — | | — | — |
| **HCV Ab positive** | Excluded | — | — | | — | | | — | — |
| **HBsAg positive** | 2.72 | 0.86, 7.27 | | **0.06** | | **3.23** | | **0.94, 9.66** | **0.045** |
| ***RISK FACTORS*** | | | | | | | | | |
| ***APOL1* genotype (ref: none)** |  | | | | |  | | | |
| One risk allele | 0.69 | 0.26, 1.71 | | 0.42 | | 0.75 | | 0.26, 2.05 | 0.58 |
| Two risk alleles | 2.16 | 0.89, 5.20 | | **0.08** | | 2.20 | | 0.83, 5.77 | 0.11 |
| **Smoking status (ref: never)** |  | | | | |  | | | |
| Current | 1.25 | 0.35, 3.46 | | 0.69 | | — | | — | — |
| Ex | Excluded | — | | — | | — | | — | — |
| **BMI (effect per 1 kg/m^2^ increment)** | 1.05 | 1.00, 1.10 | | **0.07** | | 1.04 | | 0.98, 1.11 | 0.18 |
| **Systemic hypertension** | 1.59 | 0.77, 3.43 | | 0.22 | | — | | — | — |
| **Glycaemia status (ref: normoglycaemia)** |  | | | | |  | | | |
| Pre-DM | 0.93 | 0.41, 2.06 | | 0.87 | | — | | — | — |
| DM | 0.94 | 0.33, 2.40 | | 0.91 | | — | | — | — |
| **CRP (per 1 mg/L increment)** | 1.06 | 0.99, 1.12 | | **0.08** | | 1.03 | | 0.94, 1.11 | 0.50 |

^1^Job insecurity also included as covariate. Please see Table 3.

^2^ART regimen not included as covariate.

**Table S5 (cont.)**

|  | **Lung disease** | | | | | | | | |
| --- | --- | --- | --- | --- | --- | --- | --- | --- | --- |
|  | **Univariate model** | | | **Multivariate model^1,3^** | | | **Multivariate model^2,3^** | | |
|  | ***OR*** | ***95%CI*** | ***p value*** | ***aOR*** | ***95%CI*** | ***p value*** | ***aOR*** | ***95%CI*** | ***p value*** |
| ***DEMOGRAPHICS/HIV parameters*** | | | | | | | | | |
| **Sex: Female (vs Male)** | 0.92 | 0.54, 1.59 | 0.77 | 1.01 | 0.55, 1.86 | 0.99 | 0.99 | 0.55, 1.79 | 0.96 |
| **Age (ref: <40 years)** |  | | |  | | |  | | |
| 40-50 years-old | 1.38 | 0.47, 5.03 | 0.59 | 1.17 | 0.38, 4.42 | 0.79 | 1.16 | 0.38, 4.35 | 0.81 |
| >50 years-old | 2.47 | 0.93, 8.55 | 0.10 | 1.99 | 0.71, 7.10 | 0.23 | 2.04 | 0.74, 7.27 | 0.21 |
| **Region of birth: Africa/Caribbean (vs Europe)** | 0.86 | 0.45, 1.74 | 0.66 | — | — | — | — | — | — |
| **Time since HIV diagnosis (per year)** | 1.02 | 0.98, 1.07 | 0.34 | — | — | — | — | — | — |
| **On ART** | Excluded | — | — | — | — | — | — | — | — |
| **ART regimen (ref: NNRTI-based)** |  | | |  | | |  | | |
| bPI-based | 1.29 | 0.66, 2.50 | 0.45 | — | — | — | — | — | — |
| INSTI-based | 0.86 | 0.45, 1.65 | 0.64 | — | — | — | — | — | — |
| **ART regimen (ref: TDF-based)** |  | | |  | | |  |  |  |
| Nor TDF or TAF | 1.02 | 0.55, 1.91 | 0.96 | — | — | — | — | — | — |
| TAF | 1.35 | 0.65, 2.76 | 0.41 | — | — | — | — | — | — |
| **Time on ART (per year)** | 1.02 | 0.97, 1.06 | 0.54 | — | — | — | — | — | — |
| **HIV RNA ≥200 copies/mL** | 1.49 | 0.48, 3.90 | 0.45 | — | — | — | — | — | — |
| **Nadir CD4 cell count (per two-fold increase)** | 0.99 | 0.85, 1.17 | 0.92 | — | — | — | — | — | — |
| **Recent CD4 cell count (per two-fold increase)** | 1.16 | 0.87, 1.61 | 0.34 | — | — | — | — | — | — |
| **HCV Ab positive** | 1.42 | 0.07, 9.81 | 0.76 | — | — | — | — | — | — |
| **HBsAg positive** | 0.70 | 0.16, 2.10 | 0.57 | — | — | — | — | — | — |
| ***RISK FACTORS*** | | | | | | | | | |
| ***APOL1* genotype (ref: none)** |  | | |  | | |  | | |
| One risk allele | 1.31 | 0.70, 2.47 | 0.42 | — | — | — | — | — | — |
| Two risk alleles | 1.16 | 0.51, 2.51 | 0.71 | — | — | — | — | — | — |
| **Smoking status (ref: never)** |  | | |  | | |  | | |
| Current | **2.80** | **1.24, 5.99** | **0.010** | **3.24** | **1.33, 7.53** | **0.007** | **2.70** | **1.13, 6.13** | **0.020** |
| Ex | 1.44 | 0.55, 3.31 | 0.42 | 1.44 | 0.54, 3.45 | 0.44 | 1.37 | 0.52, 3.25 | 0.49 |
| **BMI (effect per 1 kg/m^2^ increment)** | 1.02 | 0.98, 1.06 | 0.37 | — | — | — | — | — | — |
| **Systemic hypertension** | 1.45 | 0.85, 2.53 | 0.18 | — | — | — | — | — | — |
| **Glycaemia status (ref: normoglycaemia)** |  | | |  | | |  | | |
| Pre-DM | 1.59 | 0.85, 3.00 | 0.15 | — | — | — | — | — | — |
| DM | **2.41** | **1.20, 4.82** | **0.013** | — | — | — | — | — | — |
| **CRP (per 1 mg/L increment)** | 1.05 | 1.00, 1.10 | **0.06** | 1.04 | 0.98, 1.10 | 0.16 | 1.05 | 0.99, 1.11 | 0.10 |

^1^Food insecurity also included as covariate. Please see Table 3.

^2^Job insecurity also included as covariate.

^3^ART regimen and glycaemia status not included as covariate.

**Table S5 (cont.)**

|  | **Poor mental health** | | | **Chronic pain** | | |
| --- | --- | --- | --- | --- | --- | --- |
|  | **Univariate model** | | | **Univariate model** | | |
|  | ***OR*** | ***95%CI*** | ***p value*** | ***OR*** | ***95%CI*** | ***p value*** |
| ***DEMOGRAPHICS/HIV parameters*** | | | | | | |
| **Sex: Female (vs Male)** | 1.23 | 0.80, 1.90 | 0.35 | **2.47** | **1.54, 4.03** | **<0.001** |
| **Age (ref: <40)** |  | | |  | | |
| 40-50 years-old | — | — |  | — | — |  |
| >50 years-old | 0.77 | 0.37, 1.66 | 0.50 | 0.58 | 0.27, 1.25 | 0.16 |
| **Region of birth: Africa/Caribbean (vs Europe)** | 1.01 | 0.51, 2.07 | 0.98 | 0.85 | 0.43, 1.75 | 0.65 |
| **Time since HIV diagnosis (per year)** | 1.00 | 0.58, 1.77 | 1.00 | 1.22 | 0.69, 2.27 | 0.51 |
| **On ART** | 1.00 | 0.96, 1.03 | 0.80 | 1.01 | 0.98, 1.05 | 0.51 |
| **ART regimen (ref: NNRTI-based)** |  | | |  | | |
| bPI-based | 0.96 | 0.53, 1.70 | 0.88 | 0.68 | 0.38, 1.22 | 0.20 |
| INSTI-based | 1.69 | 1.02, 2.81 | **0.042** | 0.83 | 0.50, 1.39 | 0.48 |
| **ART regimen (ref: TDF-based)** |  | | |  | | |
| Nor TDF or TAF | 1.37 | 0.83, 2.27 | 0.23 | 0.96 | 0.57, 1.61 | 0.87 |
| TAF | 1.86 | 1.03, 3.37 | **0.040** | 1.37 | 0.74, 2.49 | 0.31 |
| **Time on ART (per year)** | 0.99 | 0.95, 1.02 | 0.47 | 1.01 | 0.97, 1.05 | 0.75 |
| **HIV RNA ≥200 copies/mL** | 1.25 | 0.49, 2.97 | 0.62 | 1.87 | 0.76, 4.40 | 0.16 |
| **Nadir CD4 cell count (per two-fold increase)** | 1.02 | 0.90, 1.16 | 0.82 | 0.99 | 0.88, 1.13 | 0.92 |
| **Recent CD4 cell count (per two-fold increase)** | 1.07 | 0.86, 1.35 | 0.57 | 1.01 | 0.81, 1.28 | 0.93 |
| **HCV Ab positive** | 1.54 | 0.20, 9.40 | 0.64 | 0.66 | 0.03, 4.52 | 0.71 |
| **HBsAg positive** | 0.50 | 0.16, 1.26 | 0.18 | 0.32 | 0.07, 0.93 | **0.07** |
| ***RISK FACTORS*** | | | | | | |
| ***APOL1* genotype (ref: none)** |  | | |  | | |
| One risk allele | 0.52 | 0.31, 0.85 | **0.010** | 0.75 | 0.45, 1.24 | 0.26 |
| Two risk alleles | 0.82 | 0.45, 1.46 | 0.50 | 0.77 | 0.40, 1.44 | 0.43 |
| **Smoking status (ref: never)** |  | | |  | | |
| Current | 1.22 | 0.57, 2.49 | 0.60 | 1.48 | 0.69, 3.05 | 0.30 |
| Ex | 1.42 | 0.69, 2.84 | 0.33 | 1.54 | 0.73, 3.11 | 0.24 |
| **BMI (per 1 kg/m^2^ increment)** | 1.01 | 0.98, 1.05 | 0.46 | **1.04** | **1.00, 1.07** | **0.034** |
| **Systemic hypertension** | 0.87 | 0.57, 1.34 | 0.52 | 1.48 | 0.69, 3.05 | 0.30 |
| **Glycaemia status (ref: normoglycaemia)** |  | | |  | | |
| Pre-DM | 1.17 | 0.73, 1.89 | 0.51 | 1.11 | 0.67, 1.83 | 0.68 |
| DM | 0.89 | 0.48, 1.61 | 0.70 | 1.00 | 0.53, 1.83 | 1.00 |
| **CRP (per 1 mg/L increment)** | 1.02 | 0.97, 1.06 | 0.51 | 1.05 | 1.00, 1.09 | **0.05** |

Abbreviations: ART: antiretroviral treatment. HCV Ab: Hepatitis C Virus antibody. HBsAg: Hepatitis B virus surface antigen. BMI: Body Mass Index. CRP: C-reactive protein.

ART: antiretroviral treatment. HCV Ab: Hepatitis C Virus antibody. HBsAg: Hepatitis B virus surface antigen. BMI: Body Mass Index. CRP: C-reactive protein. *APOL1*: *Apolipoprotein L1.*

**Fig S1**

**Multiple Correspondence Analysis for the main comorbidities analysed within the CKD-AFRICA study.**


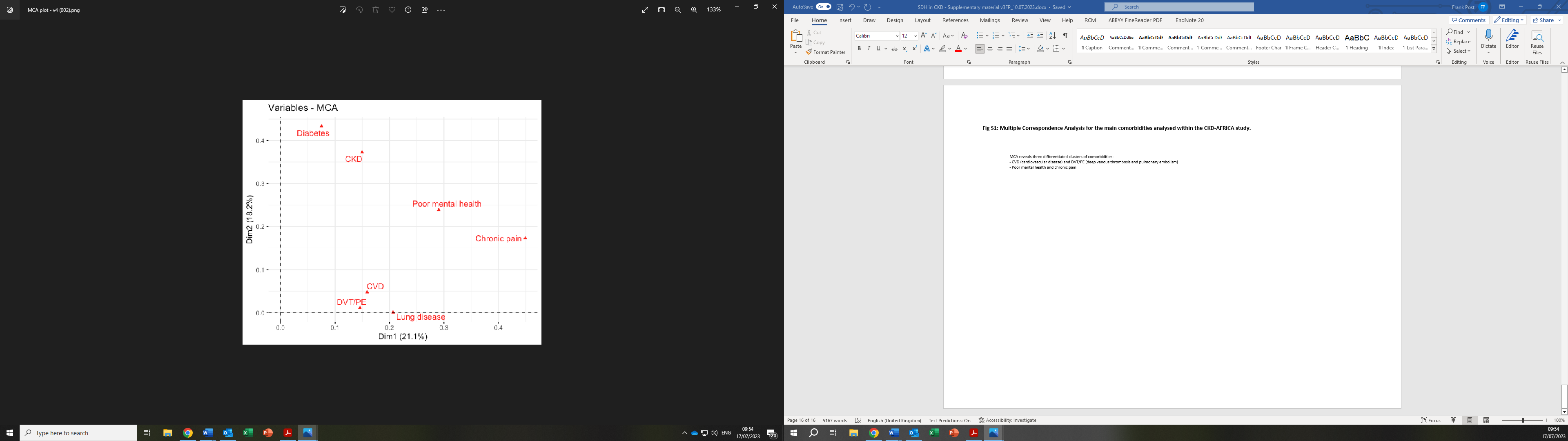


MCA reveals a degree of clustering of comorbidities:

- Diabetes and CKD (chronic kidney disease)

- CVD (cardiovascular disease), DVT/PE (deep venous thrombosis and pulmonary embolism), and lung disease

- Poor mental health and chronic pain
